# Laparoscopic based renal denervation in a canine neurogenic hypertension model

**DOI:** 10.1186/s12872-020-01546-6

**Published:** 2020-06-11

**Authors:** Chunlai Shao, Yan Zhou, Tao You, Boxin Xue, Pieter R. Stella, Ting Bo Jiang, Zhigang Miao, Longjiang Xu, Yin, Longsheng Lan, Guang Rong

**Affiliations:** 1 Department of Cardiology, The Second Affiliated Hospital of Soochow University, 1055 Sanxiang Rd., Gusu District, Suzhou, 215004 Jiangsu China; 2 Department of Urology, Anhui No.2 Provincial People’s Hospital, 1868 Dangshan Road, Hefei, 230000 Anhui China; 3 Department of Urology, The Second Affiliated Hospital of Soochow University, 1055 Sanxiang Rd., Gusu District, Suzhou, 215004 Jiangsu China; 4 Department of Cardiology, University Medical Center Utrecht, Heidelberglaan 100, 3584 CX Utrecht, the Netherlands; 5 Department of Cardiology, The First Affiliated Hospital of Soochow University, 899 Pinghai Rd., Gusu District, Suzhou, 215000 Jiangsu China; 6 Institute of Neuroscience, Laboratory Animal Center, Soochow University, 199 Ren Ai Rd. Industrial Park, Suzhou, 215123 Jiangsu China; 7 Department of Pathology, The Second Affiliated Hospital of Soochow University, 1055 Sanxiang Rd., Gusu District, Suzhou, 215004 Jiangsu China; 8 Laboratory Animal Center, Soochow University, 199 Ren Ai Rd. Industrial Park, Suzhou, 215123 Jiangsu China; 9 Department of Publicity, The Second Affiliated Hospital of Soochow University, 1055 Sanxiang Rd., Gusu District, Suzhou, 215004 Jiangsu China; 10 Department of Nuclear Medicine, The Second Affiliated Hospital of Soochow University, 1055 Sanxiang Rd., Gusu District, Suzhou, 215004 Jiangsu China

**Keywords:** Laparoscopy, Renal denervation, Refractory hypertension, Hypertension canine model

## Abstract

**Background:**

Previous renal denervation (RDN) studies showed controversial results in reducing blood pressure. The aim of this study was to provide evidence supporting the effectiveness of laparoscopic-based renal denervation (L-RDN) in treating hypertension.

**Methods:**

Sixteen Beagle dogs were randomly divided into RDN group (*n* = 12) and sham group (*n* = 4). Neurogenic hypertension was generated in all dogs via carotid artery route. L-RDN was performed in the RDN group, with sham operation performed as a control. Blood pressure (BP) changes were recorded at 2, 4, 6, and 8 weeks after the procedure. Changes in serum creatinine (sCr), blood urea nitrogen (BUN) and level of norepinephrine (NE) were analyzed. Histological changes of kidney and renal arteries were also evaluated.

**Results:**

BP and NE levels were significantly elevated after hypertension induction (*p* < 0.01). Systolic and diastolic BP of RDN group were decreased by 15.5 mmHg and 7.3 mmHg (*p* < 0.0001 and *p* = 0.0021, respectively) at the eighth week after L-RDN. Invasive systolic and diastolic BP of RDN group were significantly decreased by 14.5 mmHg and 15.3 mmHg (*p* < 0.0001). In contrast, there was no significant decrease in blood pressure in the sham group. In addition, RDN group but not the sham group showed a significant decrease in NE levels (*p* < 0.001), while no significant changes in sCr and BUN were observed in both groups. Pathological examinations showed no discernible damage, tear, or dissection to the renal arteries in RND group.

**Conclusions:**

L-RDN lowered BP and NE levels in hypertensive dogs without affecting renal artery morphology and kidney function.

## Background

In 2009, Krum and colleagues first reported the application of catheter-based renal denervation (RDN) in managing patients with refractory hypertension [1]. In a subsequent randomized controlled trial, Renal Denervation in Patients with Uncontrolled Hypertension (SYMPLICITY HTN-2) was conducted to evaluate the efficiency of RDN for controlling blood pressure in 106 patients with refractory hypertension. After a mean follow-up duration of 6 months, patients in the RDN group displayed a significant blood pressure reduction compared with the control group, and no severe complication was observed after RDN procedure [2]. These results demonstrated the feasibility and safety of this evolutionary technology in combating hypertension.

However, results obtained from SYMPLICITY HTN-3 trial in 2014 questioned the clinical application of RDN therapy. This clinical trial was rigorously designed, using randomized and controlled approaches with 535 patients diagnosed as refractory hypertension and including a sham group. The change in office systolic blood pressure (SBP) was monitored as the primary endpoint after the 6-month follow-up period. No significant difference in the reduction of office SBP was observed between RDN and sham groups by the end of follow-up [3]. These results raised a debate on the efficacy of RDN as a state-of-art therapy for hypertension and led to the suspension of RDN trials including the SYMPLICITY HTN-4 trial and the HTN trial in Japan [4].

Despite the disappointing result from the SYMPLICITY HTN-3 trial, controversial findings from different animal and human studies of RDN therapy warrant cautious interpretation. Further, clinical trials involving a diverse population from different ethnicities, as well as real-world studies with longer follow-up periods may provide comprehensive evidence on the decision to waive or retain RDN as a potential cure for refractory hypertension. Termination of RDN trials across different countries has been discouraging further clinical studies, although carefully designed preclinical studies provide an alternative approach to delineate the effect and safety of the therapy. In this study, we conducted laparoscopic radiofrequency ablation (L-RDN) of the renal sympathetic nerve from the adventitia in a hypertensive Beagle dog model and evaluated the efficiency of the modified RDN procedure.

## Methods

### Animal preparation

Sixteen male or female Beagle dogs aged 10–12 months and weighing 9.4–11.6 kg were provided and housed by the Laboratory Animal Center of the Soochow University (Jiangsu Province, China). Procedures were performed under the guidance of professional researchers in a clean laminar flow operating room. Perioperative feeding and care of the animals were carried out by professional staffs. Neurogenic hypertension was generated in all dogs, which were randomly divided into two groups, including the RDN group (*n* = 12) receiving L-RDN and the sham group (*n* = 4) receiving similar procedures except for radiofrequency ablation.

### Hypertension model induction

The neurogenic hypertension model was established via carotid artery as previously described by Jannetta et al. [5]. In all surgeries, anesthesia was induced with 3% pentobarbital sodium injection (30 mg/kg) and endotracheal intubation. If the operation last more than 2 h, 3 mg/kg pentobarbital sodium should be added every half hour. After successful anesthesia and conventional disinfection, a vertical cervical incision was performed to the left side of the midline to allow sequential separation of the skin, muscles, and connective tissue layers. After full exposure of the left common carotid artery (LCA) and isolation of the vagal trunk, 4–0 enteric chromium surgical suture was cut into 1-2 mm fragments and placed lightly around the vagus nerves. The vagus nerve and surrounding connective tissue were fixed to form cross compression between the vagus nerve and the LCA, and the regular pulse of the carotid artery formed continuous compression on the vagus nerve.

### L-RDN procedures

**Fig. 1:**
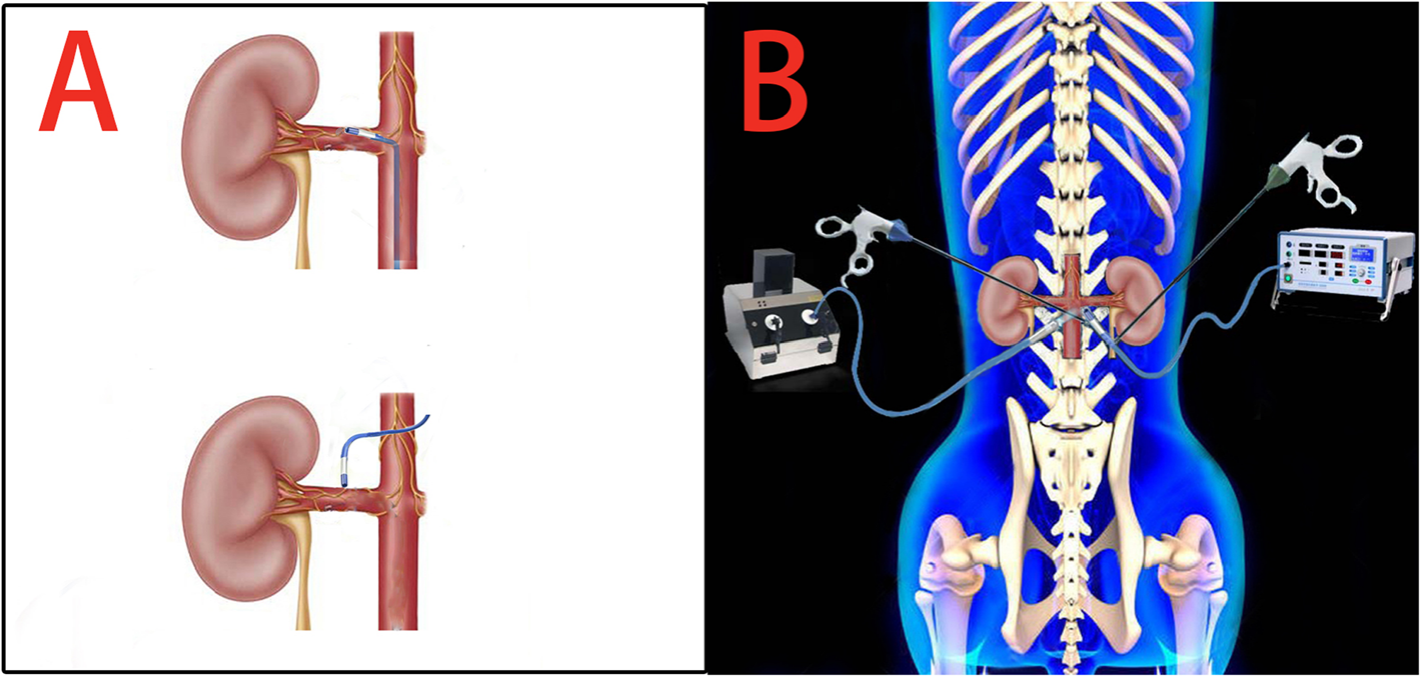
Schematic of L-RDN:**a** The different ways of Endovascular ablation (catheter way) and Extra vascular ablation (laparoscopic way). **b** Radiofrequency catheter directly ablation the renal artery adventitia sympathetic nerve under the assistance of laparoscopic system

After successful anesthesia, each animal in the RDN group was placed in a supine position with head and limbs secured on an operating table. The abdominal skin was prepared by shaving and disinfection. A 1-cm midline abdominal incision was made at 3 cm below the umbilicus. A double-channel trocar of 10-mm in diameter was inserted, through which an operative laparoscope (Karl Storz, Tuttlingen, Germany) was introduced. Carbon dioxide pneumoperitoneum of 10–12 mmHg was generated. Two additional 5-mm trocars were inserted through incisions in both midaxillary lines at 15 cm above the anterior iliac crest. Another two 5-mm trocars were inserted at the middle points between the laparoscope trocar and the anterior iliac crest trocars. The laparoscopic hook electrode, suction and elastic separating plier were inserted as appropriate. A combination of blunt and sharp dissection of the renal fascia was applied until the renal hilus was visualized clearly. The radiofrequency ablation catheter (7F IBI radiofrequency ablation catheter; St. Jude Medical) was inserted through the side port of the double-channel trocar towards the renal artery and connected to a radiofrequency ablation device (IBI, St. Jude Medical, Inc., St. Paul, MN, USA) (Fig. 1a, b). Six to eight ablation sites were selected from bilateral trunks of renal arteries through branches. Each spot was ablated for 60 s, with a power limit of eight W, until the vascular adventitial temperature reached 55 °C. During the procedure, we ablated bilateral renal arteries from the proximal end to the distal end of the renal arterial trunk. After ablation of all points, laparoscopic devices were removed from the abdominal cavity and incisions were closed layer by layer with sutures. Each dog was given 1.6 × 10^6^ units of penicillin i.m. postoperatively for three consecutive days. Dogs in the sham group underwent similar laparoscopic surgery except for radiofrequency ablation.

### Blood pressure monitoring

Non-invasive blood pressure (NIBP) was measured using a blood pressure cuff around one thigh of the hind limbs and connected to a multifunctional blood pressure monitor. NIBP were monitored before hypertension induction (defined as the baseline) and 2, 4, 6, and 8 weeks after hypertension induction. At the eighth week after hypertension induction, L-RDN and sham surgeries were performed in the RDN group and sham group, respectively, and changes in NIBP were recorded at 2, 4, 6, and 8 weeks after the procedures.

To measure invasive blood pressure (IBP), animals were anesthetized and the right femoral area was disinfected. After successful puncture with a syringe, a pressure transducer (TruWave PX260, Edwards Lifesciences, USA) was attached for temporal IBP measurement using a PHILIPS IntelliVue X2 System. IBP was monitored before hypertension induction (defined as the baseline), at 8 weeks after hypertension induction, and at 8 weeks after RDN or sham procedures.

### Renal function and norepinephrine levels determination

The renal functions and norepinephrine (NE) levels before hypertension induction (defined as the baseline), at 8 weeks after hypertension induction, and at 8 weeks after RDN or sham procedures were measured at the Institute of Neuroscience and Laboratory Animal Center of Soochow University. All Beagle dogs were humanely killed by intravenous injection of potassium chloride solution (2 mg/kg) under anesthesia at 8 weeks after RDN.

### Histological analysis and immunofluorescence staining

All experimental animals were humanely killed 8 weeks after RDN to isolate the complete renal arteries and kidneys for gross specimen examination for evidence of stenosis, dissection, and perforation in renal arteries. Tissue specimens were fixed in 4% paraformaldehyde, followed by paraffin-embedding, tissue sectioning, and hematoxylin and eosin (HE) staining using a commercial kit (C0105, Beyotime, Shanghai, China). Light microscopy was used to evaluate the histomorphological changes of the renal arteries in different groups.

### Immunofluorescence staining

Transverse sections of the renal artery (15 μm, Leica Cryostat CM3050) were collected directly in glass slides (4–8slides/tissue, 2–4 slices/slide) and incubated with a blocking buffer containing 10% goat serum, 1% bovine serum albumin (BSA), and 0.3% Triton X-100 for 1 h. The sections were then incubated with anti-VEGF antibody (1:1000, Abcam, USA) at 4 °C overnight. After washing with PBS for 3 times, the sections were incubated with fluorescent-conjugated anti-rabbit secondary antibodies (11,000, Jackson ImmunoResesrch Laboratories, PA, USA) and counterstained with DAPI for 1 h. All slides were examined under a fluorescence microscope (AXIO SCOPE A1, ZEISS, Germany).

### Statistical analysis

Statistical analysis was performed using SPSS software (version 22, SPSS Inc., Chicago, Illinois, USA). Continuous variables with normal and skewed distribution were expressed as means with standard deviations or medians with interquartile ranges. Categorical variables were presented as numbers and percentages. Changes in BP were expressed as mean (95% CI). Independent samples t-test and Mann-Whitney U test were used to compare the differences in continuous variables with normal and skewed distribution between RDN and sham groups. Comparison of continuous variables with normal and skewed distribution at different time points in the same group was performed using paired t-test and Mann-Whitney U test, respectively. Categorical data between the groups were compared using the Chi-square test. Homogeneity-of-variance was determined by Levene’s test of equality of variances. A two-way repeated measures ANOVA (blood pressure × time after the procedure) adjusted for baseline blood pressure was performed using the BP reduction as dependent variables. Sphericity was determined by Mauchly’s test, and interactions between variables were estimated. Variables were compared using the Greenhouse-Geisser adjustment method when sphericity was absent. Otherwise, comparisons were performed using the simple effects tests if interactions were present, or using the tests of within-subjects effects if no significant interactions were observed. Bootstrapping (1000 replications) with a simple percentile 95% CI was used to validate the results. A *p*-value < 0.05 was considered as statistically significant.

## Results

### L-RDN reduces BP in animals with neurogenic hypertension

**Fig. 2:**
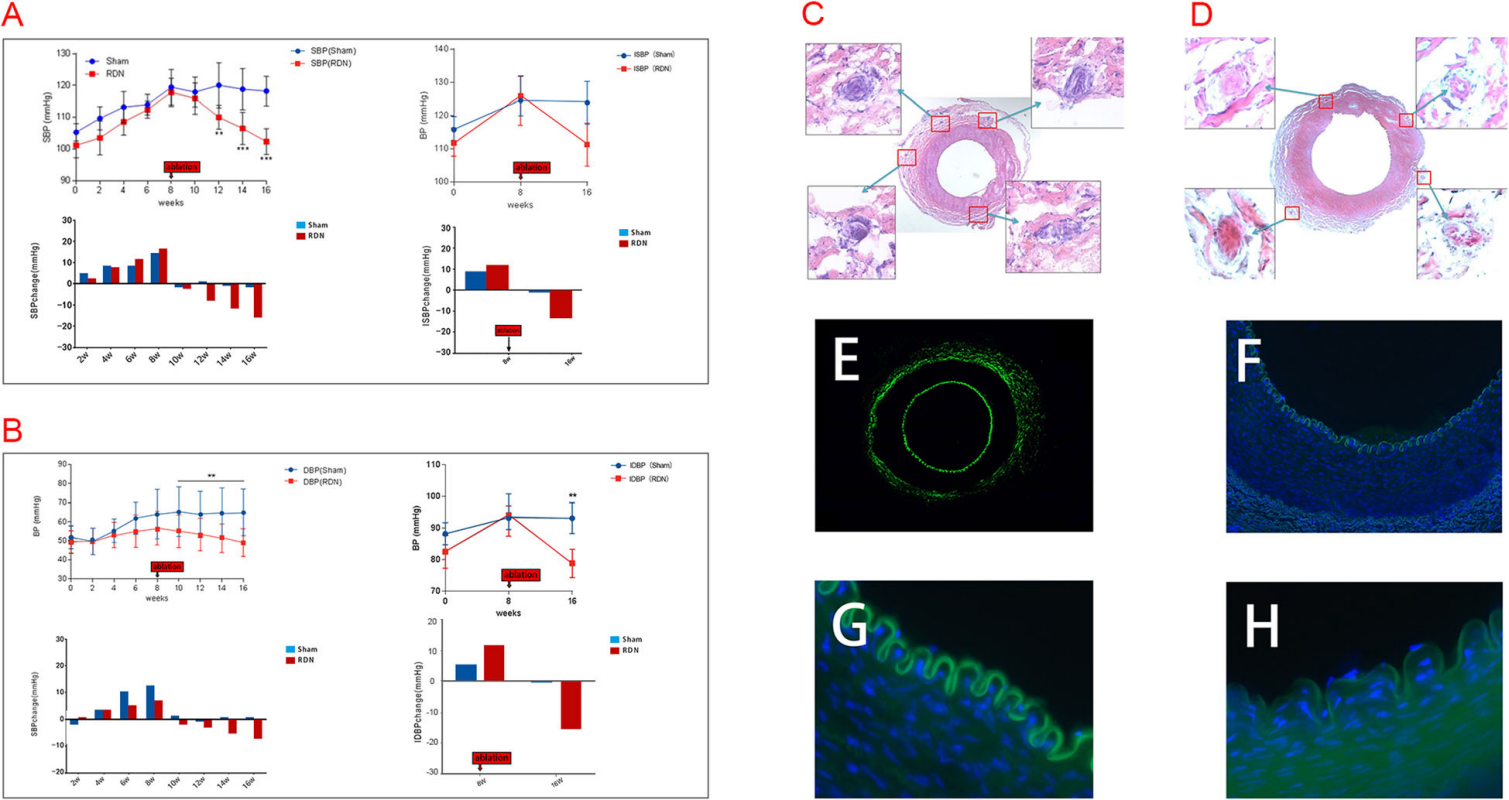
**a** and **b**: Changes in blood pressure (SBP, DBP, ISBP and IDBP) at pre- and post-induction of hypertension and post- procedure of hypertension. SBP = systolic blood pressure. DBP = diastolic blood pressure. ISBP = invasive systolic blood pressure. IDBP = invasive diastolic blood pressure. **P < 0.01, ****P < 0.0001.Histological Examination of the Target Nerve Bundles (**c**) 8 weeks after Neurogenic hypertension modeling in sham group (H&E × 100, × 400). Photomicrograph of a histologic cross-section of complete renal sympathetic nerve tissue was shown before RDN. (**d**) 8 weeks after ablation in RDN group (H&E × 100, × 400). The renal artery with intact wall and endothelium was surrounded by the damaged nerve fibers. The nerve fibers show coagulation necrosis and absence of nuclei. Fluorescent immunostaining shows the renal artery with intact wall and endothelium in RDN group at 8 weeks after ablation procedure. (E × 50, F × 100, G × 200, H × 400)

During 8 weeks after neurogenic hypertensive induction, a continuous elevation of BP in animals was observed in all dogs (Table 1 and Table 2). Compared with the peak BP recorded by 8 weeks after hypertension induction, SBP and DBP in the RDN group were decreased by 15.5 mmHg and 7.3 mmHg at the eighth week after the procedure (*p* < 0.0001 and *p* = 0.0021) (Table 3). Consistently, L-RDN significantly decreased ISBP and IDBP of animals by 8 weeks after the procedure by 14.5 mmHg and 15.3 mmHg (*p* < 0.0001) (Table 4). In contrast, no significant changes in BP and IBP were observed in the sham group throughout 8 weeks after the sham procedure (Table 3 and Table 4). The overall changes in BP were displayed in Fig. 2a and b. Given the presence of a significant interaction between time (weeks after the procedure) and treatment (RDN or sham), we evaluated the simple effects of the treatment. In the RDN group, higher reductions in SBP from 4 weeks and DBP from 6 weeks after the L-RDN procedure were observed compared with the sham group (Table 3). Similar increments in IBP reduction were achieved by L-RDN compared with the sham procedure (Table 4).

**Table 1:**
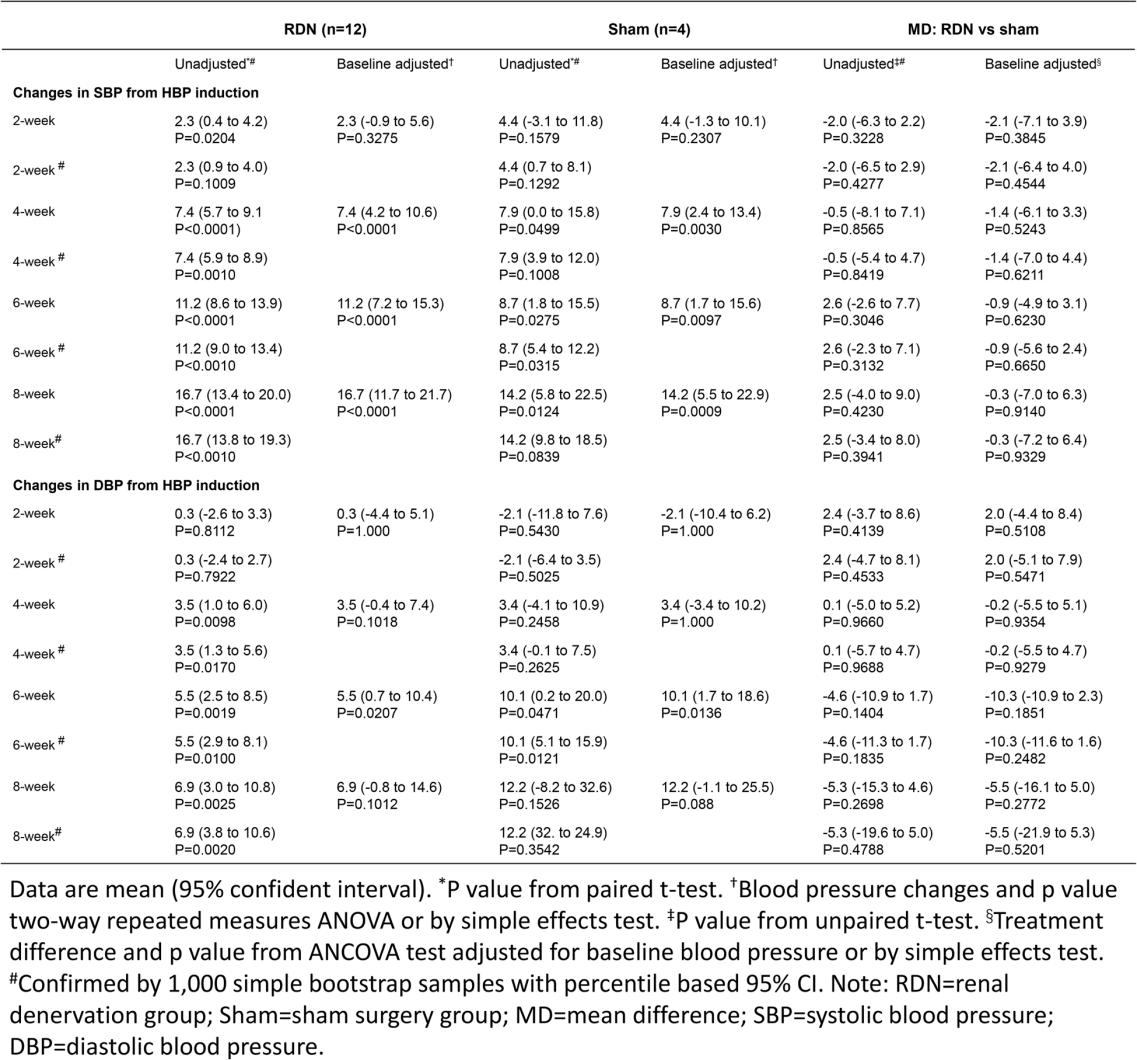
Changes in blood pressure for 8 weeks form hypertension induction. Data re mean.

**Table 2:**
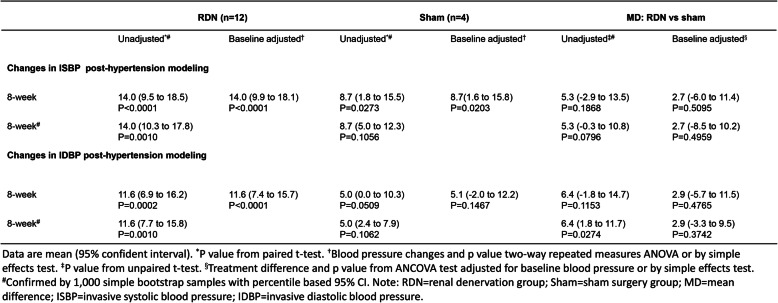
Changes on invasive arterial blood pressure for 8 weeks post-hypertension modeling.

**Table 3:**
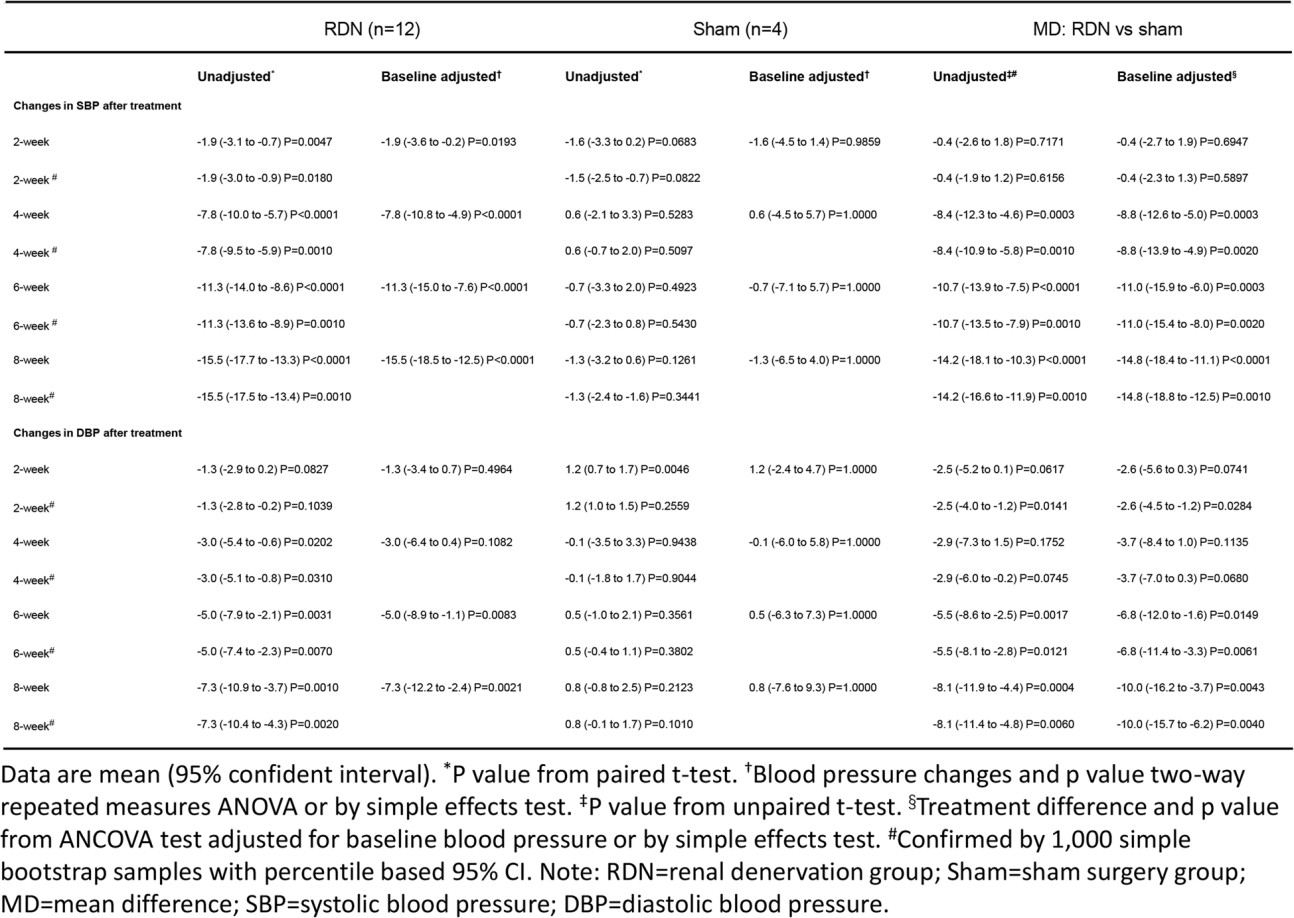
Changes in blood pressure through 8 weeks after the procedure.

**Table 4:**
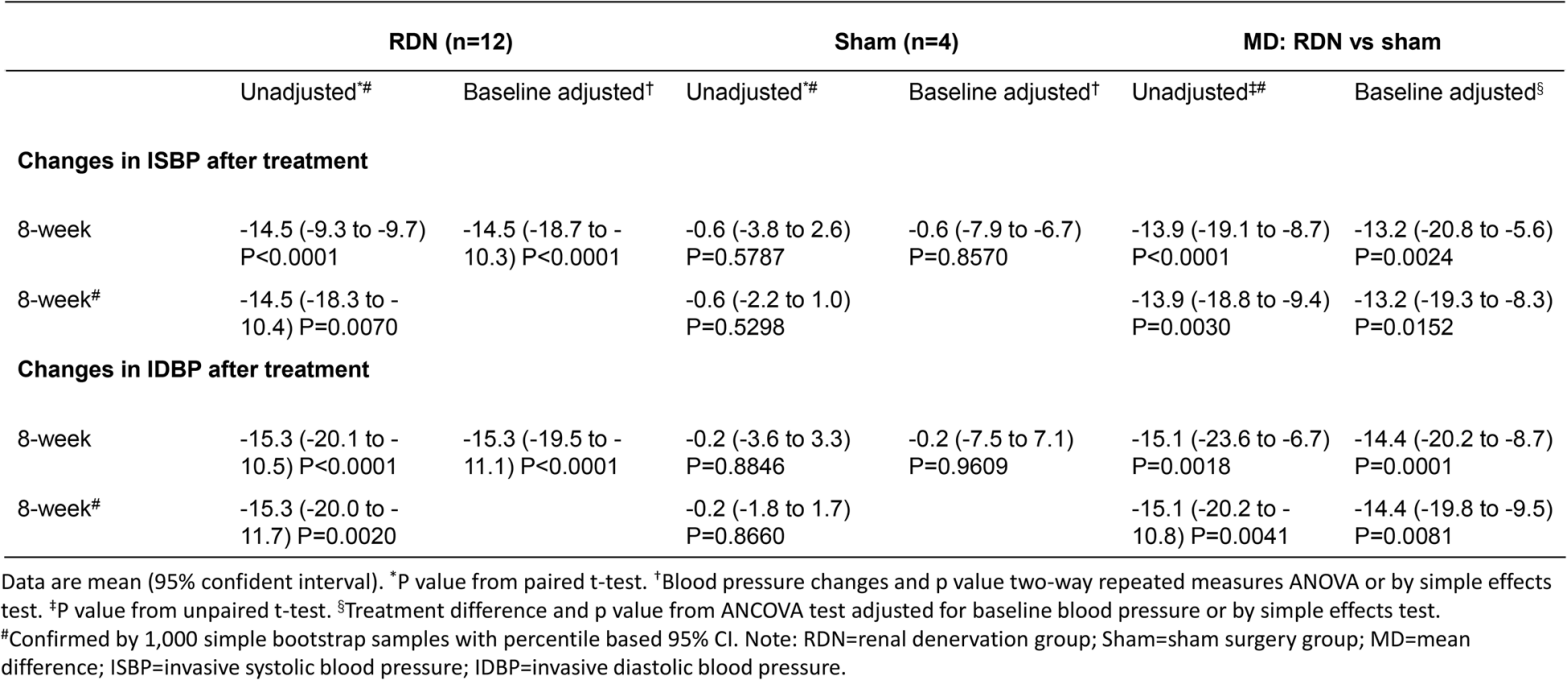
Change in invasive arterial blood pressure through 8 weeks after the procedure.

### Comparison of changes in plasma NE and renal functions between RDN and sham group

Circulating NE levels in dogs from RDN and sham groups were elevated after hypertension induction and peaked at the eighth week compared with the baseline levels (*p* = 0.002, *p* = 0.001). Eight weeks after the radiofrequency ablation, NE levels of the RDN group were reduced from the preoperative level of (554.6 ± 36.3) μg/L to a baseline level of (354.9 ± 59.7) μg/L (*p* = 0.0011). In contrast, the sham group showed no significant difference in plasma NE levels by the end of follow-up (*p* = 0.948) (Table 5). In addition, both groups displayed no significant changes of BUN and sCr in 8 weeks after hypertension induction, as well as from RDN or sham surgery to the end of follow-up (Table 5).

**Table 5:**
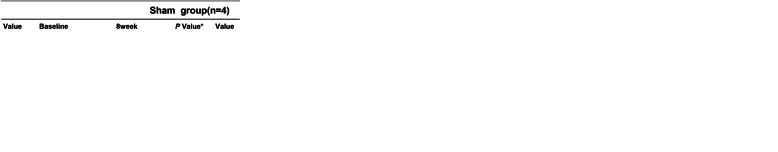
Changes in sympathetic activity detection and renal functions.

### Comparison of histological changes in the renal artery of animals between RDN and sham group

HE stains revealed three intact concentric layers of the renal artery, including the intima, media, and adventitia in the sham group. The intima was composed of elastic fibers and endothelial cells with continuous elastic fibers. The media was composed of 10 to 30 circular and orderly-arranged smooth muscle layers with elastic fibers and collagen fibers. These histological findings remained unchanged by 8 weeks after hypertension induction of (Fig.2c). On the other hand, the adventitia in the RDN group by 8 weeks after L-RDN was composed of loose connective tissue occasionally scattered by nerves tracts with coagulation necrosis, feature by digestion chambers, vacuolization, nuclear pyknosis, and significant loss of nuclear and cellular components (Fig.2d).

### Safety of L-RDN procedure

Under different magnification conditions, we evaluated the damage on intima of renal artery 8 weeks after L-RDN. VEGF was present in the arterial intima where measurements were made. There was obvious spontaneous fluorescence in the intimal surface of blood vessels. The results showed that vessel wall structure were normal without any perivascular cellular infiltrates, vessel wall thickening, dissection and other pathological findings by 8 weeks after L-RDN (Fig. 2e, f, g and h).

## Discussion

Despite the rapid development of novel anti-hypertensive regimes, refractory hypertension remains a major challenge in controlling blood pressure in certain patient groups. In the 1930s, excision of visceral sympathetic ganglia had been proposed as a treatment strategy in severely hypertensive patients [6]. However, the surgical approaches may result in postoperative mortality and long-term complications, including orthostatic hypotension, rectum, and bladder sphincter dysfunction, and erectile dysfunction [7]. In recent years, with the development of interventional therapy-related devices, a novel type of catheter-based and point-to-point renal sympathetic denervation procedure has been used to achieve the eradication of sympathetic nerves and sustained reduction of BP [8]. Krum and colleagues administrated RDN to 45 patients with refractory hypertension and significantly reduced average BP by a maximum of 27/17 mmHg in 12 months after the procedure [1]. Later, results of a large-scale randomized controlled multicenter clinical trial, SYMPLICITY HTN-2, confirmed the efficacy and safety of RDN, spurring great interest in the technology among cardiovascular clinicians [2]. However, with the publication of negative results from the more stringent SYMPLICITY HTN-3 trial that failed to show the significance of RDN in reducing BP, RDN suddenly lost favor and was discontinued in most hospitals [3]. Several reasons have been proposed to underlie the negative results of SYMPLICITY HTN-3 trial. First, even stringent inclusion criteria for patients receiving catheter-based RDN may not be reasonable. It was unclear whether all refractory hypertension cases were due to excessive excitation of the renal sympathetic nerve, therefore some patients with pathogenesis beyond renal sympathetic hyperactivity might not respond well to catheter-based RDN. Although the catheter-based RDN aimed to inhibit the excessive excitation of renal sympathetic nerve, the activity of renal sympathetic nerve were not determined in this trial either at enrollment or after the RDN procedure [9]. Second, the renal nerve ablation systems may need further improvement. The SYMPLICITY catheter-based system used in SYMPLICITY HTN-3 trial, derived from the widely used conventional intravascular renal nerve ablation system, may be compromised by several drawbacks. For instance, the single electrode only permits ablation of a single point each time thus requires extended operating time to complete the procedure. The tip of the device could hardly adhere to the vascular wall due to the tortuous shape of renal arteries. Thus, it was difficult to ensure a 360° spiral ablation on the entire renal arterial wall. The low power of radiofrequency energy led to limited penetration depth and ablation efficacy of the sympathetic nerve despite safety benefits from less vascular damage [10]. Third, surgical experience of surgeons who performed RDN might have affected the results of the trial [11]. Fourth, the anatomy of renal sympathetic nerve may render a higher energy requirement for successful ablation. The majority of sympathetic nerve fibers course along the vascular adventitia into the renal parenchyma and exhibit a network-like distribution in the adventitia and adjacent tissue. Sympathetic nerves and ganglia are more abundantly distributed in proximal renal artery with an average depth over 5 mm from the arterial luminal side [12, 13]. As the average ablation depth of available RF RDN systems reaches 3 to 4 mm, intravascular RDN might not be sufficient to ablate the proximal sympathetic nerves [14].

In this study, the L-RDN of sympathetic nerves located in the renal artery adventitia had several anatomical advantages [15]. The procedure was not achieved using the intraluminal ablation and the catheter could directly contact the sympathetic nerve of the renal artery adventitia (Fig. 1). This allowed the surgeon to selectively ablate sympathetic nerves according to their distribution pattern and maximize the ablation efficiency without damaging the intima of the renal artery. Moreover, it also permitted effective RDN with multiple renal arteries or even malformation of the renal artery. Hence, the L-RDN approach used in this study may benefit from increased ablation accuracy, energy utilization efficiency, and operational flexibility. In addition, the entire procedure was completed without referring to X-ray exposure and may ensure the safety to both surgeons and patients compared with conventional ablation procedures. It should be noted that some studies have begun to try to ablate sympathetic nerves from the renal artery adventitia pathway in recent years. Wang et al. used non-invasive high-energy focused ultrasound to ablate the renal sympathetic nerve in an animal model and significantly reduced blood pressure [16]. In 2016, a 59-year-old woman with blood pressures as high as 220/110 mmHg underwent bilateral laparoscopic renal denervation. Finally, the patient’s blood pressure was reduced to the range of 120–140/80–90 mmHg at the 1-, 3-, 9-, and 12-month follow-ups [17]. Ye E et al. designed a novel laparoscopy-based renal denervation (L-RDN) system with a looped bipolar electrosurgical instrument. They evaluated the thermal effectiveness via simulation study on a numerical model designed using histological data and validated the results by the in vitro study. Finally, the simulation results showed that new device is safer and more effective than the existing catheter-based systems [18]. These studies show that RDN from renal artery adventitia pathway is feasible in the treatment of hypertension, which also supports our results.

In this study, Beagle dogs in the RDN group received radiofrequency ablation after successful induction of hypertension and had a significant reduction in postoperative BP. Blood pressure was reduced to baseline level by 8-week after L-RDN, indicating that L-RDN completely reversed the hypertension effect of neurogenic insults. Moreover, the RDN group had a significant reduction in plasma NE levels, suggesting that L-RDN lowered the sympathetic nerve activity [19]. These results support L-RDN as a candidate for controlling refractory hypertension caused by neurogenic pathogenesis.

HE staining of the postoperative renal arteries showed that the sympathetic nerve tracts in the sham group was wrapped by a thin layer of fibrous connective tissue and remained intact. In contrast, the fibrous connective tissues surrounding the sympathetic nerve tracts of renal arteries in the animals of RDN group were thickened, with connective tissue replacing the nerve tracts and membranes. No obvious smooth muscle proliferation and inflammatory cell infiltration were observed in renal arteries from dogs of both groups. In addition, renal arterial intima and media in both groups were free of damage, tear, or dissection, with no renal atrophy, necrosis, and other complications being observed. These results suggest that the major form of renal sympathetic nerve damage achieved by ablation was nerve fibrosis. These results were consistent with the changes in different layers of renal arteries associated with radiofrequency ablation in previous in vivo studies [20, 21]. The safety of L-RDN was reinforced by unchanged sCr and BUN in the Beagle dogs after RDN. In addition, gross anatomy examination displayed no renal artery stenosis, dissection, or other complications after L-RDN. Taken together, these findings support the safety of L-RDN as a potential anti-hypertensive therapy.

The present study demonstrated that the ablation of the sympathetic nerve in the renal artery adventitia effectively reduced BP in a hypertensive animal model. The combination of Laparoscopy and RDN constitutes a new approach with enhanced therapeutic efficiency. Recently in Lancet, Townsend et al. reported the results of a randomized, sham-controlled, proof-of-concept trial named catheter-based renal denervation in patients with uncontrolled hypertension in the absence of antihypertensive medications (SPYRAL HTN-OFF MED). In this study, renal denervation significantly lowered BP in patients with untreated mild to moderate hypertension. Compared with the sham group, 80 patients in the experimental group achieved average reductions in ambulatory SBP by five mmHg and in DBP by 7.7 mmHg, provided the latest evidence supporting the effectiveness of RDN therapy [22].

Our results demonstrated adventitial ablation as an alternative pathway that allows safe and efficient ablation of the renal sympathetic nerve. Further animal studies are warranted to evaluate L-RDN in different models of hypertension before proceeding with human studies with more stringent selection criteria on specific patient groups. Quantitative assessment of the renal sympathetic nerve activity and improved devices may also facilitate future clinical studies of RDN that may hopefully provide a novel avenue in the treatment of refractory hypertension.

## Conclusions

In this study, the effectiveness of randomized RDN therapy was evaluated using a canine model of surgically induced neurogenic hypertension. Compared with sham surgery, laparoscopic renal denervation significantly lowered both systolic and diastolic BP. Meanwhile, L-RDN reduced NE levels while not altering sCr and BUN. Renal artery pathology showed no obvious vascular damage after L-RDN. In conclusion, L-RDN could be a useful treatment to optimize blood pressure control.

### Study limitations

This study modeled neurogenic hypertensive animals through the carotid artery that might not recapitulate the pathogenesis of human neurogenic hypertension. In addition, we only monitored changes in BP and circulating NE levels to evaluate the therapeutic efficacy of radiofrequency ablation due to the lack of direct assessment of renal sympathetic nerve activity in the in vivo models. In addition, our study had a small sample size and the short-term BP monitoring (i.e. 8 weeks). The main limitation is that this is just an animal experiment, and its positive results may not be achieved in human clinical treatment. Since this surgical approach is expected to treat hypertensive patients, large-scale animal studies and clinical trials are warranted to further investigate the anatomical routes for laparoscopy, energy range, duration, and equipment of radiofrequency ablation.

## Data Availability

The data and materials of this article are included within the article.

## Abbreviations

RDN: Renal denervation
L-RDN: Laparoscopic based renal denervation
BP: Blood pressure
sCr: Serum creatinine
BUN: Blood urea nitrogen
NE: Norepinephrine
SBP: Systolic blood pressure
DBP: Diastolic blood pressure
LCA: Left common carotid artery
NIBP: Non-invasive blood pressure
ISBP: Invasive systolic blood pressure
IDBP: Invasive diastolic blood pressure
HE: Hematoxylin and eosin
BSA: Bovine serum albumin
